# Applicability of NMR spectroscopy to quantify microplastics across varying concentrations in polymer mixtures†

**DOI:** 10.1039/d5ra01174d

**Published:** 2025-04-23

**Authors:** Julia Schmidt, Marte Haave, Wei Wang

**Affiliations:** a Department of Chemistry, University of Bergen 5007 Bergen Norway wei.wang@uib.no; b Centre for Pharmacy, University of Bergen 5020 Bergen Norway; c SALT Lofoten AS 8301 Svolvær Norway

## Abstract

Quantitative nuclear magnetic resonance (qNMR) spectroscopy could potentially be used for environmental microplastic analyses, provided the challenges posed by mixed polymer samples with varying concentrations and overlapping signals are understood. This study investigates the feasibility of qNMR as a reliable and cost-efficient method for quantifying synthetic polymers in mixtures of low and varying concentrations, addressing key challenges and limitations. Polymer mixtures were analysed using deuterated chloroform (CDCl_3_) and deuterated tetrahydrofuran (THF-d_8_) as solvents, with polystyrene (PS), polybutadiene-cis (PB), polyisoprene-cis (PI), polyvinyl chloride (PVC), polyurethane (PU), and polylactic acid (PLA) as selected polymers. Mixtures contained either low and high concentrations of each polymer or equal concentrations of all six polymers. Polymer concentrations were measured using the internal standard method. The method showed low relative errors for low concentrations of PS in CDCl_3_ and PVC in THF-d_8_, with values of −5% and 0%, respectively, while PB and PI in CDCl_3_ show relative errors of +5% and −3%, respectively. We observe significant linearity between nominal and measured concentrations with *R*^2^ values ranging from 0.9655 to 0.9981, except for PU, which had high relative errors and poor linearity (*R*^2^ = 0.9548). Moreover, simultaneous quantification of six polymers in THF-d_8_ proves effective at intermediate concentrations. However, overlapping proton signals are observed, causing high-concentration polymers to mask low-concentration ones. While this study demonstrates low limit of quantification (LOQ) and advances in simultaneous polymer quantification, further research is needed to improve qNMR accuracy for mixed polymer samples and environmentally relevant concentrations.

## Abstract 2

qNMR spectroscopy is evaluated for quantifying synthetic polymers in mixtures. Overlapping signals and low concentrations challenge its application in environmental samples, but intermediate levels show promising results.

## Graphical abstract

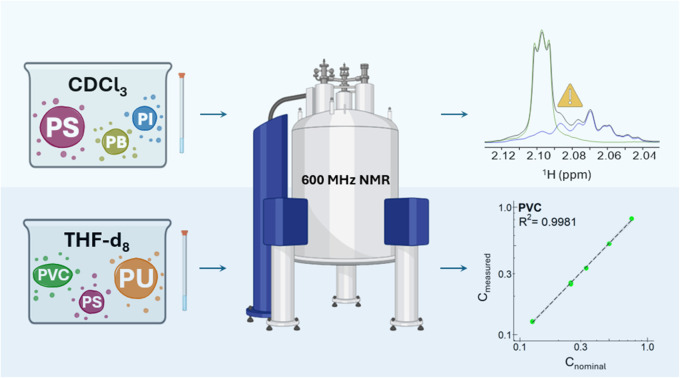

## Introduction

Plastic products are ubiquitous in modern society,^1–3^ which has led to increasing global concern. Correspondingly, microplastics (MP, 1 μm–5 mm)^2,4^ and nanoplastics (NP, <1 μm)^5–8^ have emerged as major environmental issues due to their potential risks to human health and the environment.^3,9^ The widespread presence, small size, and ability of MPs to be taken up and translocated into biological tissues make them a critical environmental concern,^10–12^ necessitating further research on their toxicity.^4,13,14^

Despite growing awareness, there are no ISO standards for MP quantification, and current methods remain inadequate. Existing approaches include optical methods such as scanning electron microscopy (SEM), Fourier transform infrared spectroscopy (FTIR), and Raman spectroscopy,^15,16^ as well as thermoanalytical techniques such as thermal desorption gas chromatography-mass spectrometry (TDS-GC-MS) and pyrolysis GC-MS.^17–19^ Novel techniques like laser-induced breakdown spectroscopy (LIBS) and laser ablation inductively coupled plasma mass spectroscopy (LA-ICP-MS) also show promise in identifying aged microplastics.^20^ However, these methods are often costly, time-consuming, and typically involve single replicate analysis due to high sample costs.^21^ Therefore, fast, reliable, and cost-effective polymer quantification is essential for environmental risk assessments.

In recent years, proton nuclear magnetic resonance (^1^H NMR) and quantitative NMR (qNMR) spectroscopy has become a popular method for detecting and analysing MP particles^22–27^ due to its rapid, cost-effective, and precise nature.^28,29^ However, challenges exist, including the need for suitable solvents to dissolve polymers, the loss of particle size, colour, and shape information, and the requirement for high temperatures to dissolve resistant polymers like polypropylene (PP) and polyethylene (PE).^30–32^ Recent research has focused on analysing various polymers, such as low-density polyethylene (LDPE), polystyrene (PS), polyethylene terephthalate (PET), acrylonitrile-butadiene-styrene (ABS), polyamide (PA), polyvinyl chloride (PVC), polyurethane (PU), polylactic acid (PLA), polybutadiene (PB), polyisoprene (PI), polymethylmethacrylate (PMMA), and polyacrylonitrile (PAN), offering insights into the proton signals of pure polymer.^22–24,27,33^ This first step towards differentiating various proton signals of polymers provides a basis for the quantification of polymers in mixtures.

While some previous research has explored the application of qNMR spectroscopy to polymer mixtures at high or uniform concentrations,^33,34^ the challenge of quantifying low and varying concentrations, reflecting the complexity of environmental samples, remains unaddressed. This study therefore investigates and describes the challenges and potential pitfalls in quantifying mixed polymer samples at low and varying concentrations. We report uncertainties and relative errors when quantifying polymer mixtures, using concentrations close to the previously determined limit of quantification (LOQ) for single polymers.^27^ Additionally, we assess whether the precision in the analyses changes in polymer mixtures with differing concentrations due to overlapping or interfering proton signals. This is a highly necessary step towards understanding the reliability and limitations of qNMR spectroscopy for real environmental sample analysis (Schmidt *et al.*, in prep).

## Experimental

### Materials

Commercially available polymer particles of polystyrene (PS), polylactic acid (PLA), and polyurethane (PU) beads (3–5 mm) from GoodFellow Cambridge Ltd, England; polyvinyl chloride (PVC) powder (<50 μm, > 99.7% purity) from Werth-Metall, Germany, natural rubber polyisoprene-cis (PI), and synthetic polybutadiene-cis (PB) from Sigma-Aldrich®, were utilized as model particles. As solvents, deuterated chloroform (CDCl_3_, 99.8 atom % D) from Sigma-Aldrich® with its residual proton signal at 7.26 ppm and deuterated tetrahydrofuran (THF-d_8_, ≥99.5 atom % *D*) purchased by Sigma-Aldrich® and VWR International, LLC with its residual proton signals at 3.58 ppm and 1.73 ppm, were used. Dimethyl sulfone (DMSO_2_) purchased by *Trace*CERT®, Sigma-Aldrich® with its proton signal at 3.00 ppm was utilized as an internal standard.

### Sample preparation

This study consists of three parts. The first part involves acquiring a single ^1^H NMR spectrum of the polymers PS, PB and PI in CDCl_3_ and PS, PVC and PU in THF-d_8_. A high concentration (1 mg mL^−1^) was chosen and served as quality control and reference for the polymer mixtures in the setups described below. For the second part, all polymer mixtures were prepared from a stock solution with a high concentration. The first setup (Table 1) consists of a mixture of three polymers of equally high concentrations (333 μg mL^−1^). PS, PB and PI were dissolved in CDCl_3_ (setup 1A) and PVC, PU, PS in THF-d_8_ (setup 1B). Setup 2A consists of mixtures of three polymers in CDCl_3_, always including one high and two low concentrations (Table 1, Mixture 1–3). Setup 2B used mixtures of three polymers in THF-d_8_, always including one low and two high concentrations (Table 1, Mixture 4–6). The lower concentrations used in setups 2A and 2B, were the LOQ for that polymer.^27^ The third setup consisted of sets of six mixtures in CDCl_3_ or THF-d_8_ with concentration gradients that made up a calibration curve (Table 1, setup 3A and 3B). These mixtures served as calibration samples. The calibration concentration levels were 750, 500, 333, 250 and 125 μg mL^−1^. The concentrations 125 and 250 μg mL^−1^ of each polymer were used twice in different constellations with other polymers, while the concentrations 500 and 750 μg mL^−1^ were represented once per polymer. The third part involved preparing a polymer mixture in the fourth setup, consisting of equal parts of PS, PVC, PU, PI, PB and PLA in THF-d_8_. Each polymer had a nominal concentration of 166 μg mL^−1^.

**Table 1:** Overview of the four experimental setups of polymer mixtures in CDCl_3_ or THF-d_8_^a^

	Setup	PS	PB	PI	PS	PVC	PU
		Nominal concentration [μg mL^−1^]					
**Part 2**	**Solvent**	**CDCl**_**3**_ **(A)**			**THF-d**_**8**_ **(B)**		
	Setup 1A and 1B	333	333	333	333	333	333
	Setup 2A and 2B	1000	0.4	0.6	4	100	100
		4	1000	0.6	200	8	100
		4	0.4	1000	200	100	6
	Setup 3A and 3B	500	250	250	500	250	250
		250	500	250	250	500	250
		250	250	500	250	250	500
		750	125	125	750	125	125
		125	750	125	125	750	125
		125	125	750	125	125	750

**Part 3**	**Solvent**	**THF-d**_**8**_ **(B)**					
	Setup 4	166	166	166	166	166	166

a PS = polystyrene, PB = polybutadiene-cis, PI = polyisoprene-cis, PVC = polyvinyl chloride, PU = polyurethane.

All polymers in their corresponding solvents were dissolved at room temperature. To quantify the polymers, present in the sample, the internal standard of DMSO_2_ was prepared in both solvents and added to all setups with a known concentration. For the NMR measurements, each sample solution (600 μL) was transferred into 5 mm NMR tubes (Bruker BioSpin, 4′′ NMR tubes) and subsequently analysed.

### qNMR

All NMR experiments were conducted using a Bruker Ascend 600 MHz spectrometer equipped with an AVANCE NEO console and a QCI-P CryoProbe™. All measurements were performed at a temperature of 298 K. For the qNMR measurements, the acquisition parameters were standardized across all polymer mixtures, with the pulse width and receiver gain automatically calibrated for each sample. The spectral width was set to 29.76 ppm, the number of scans was 8, the spectral size was 262 144 points, the acquisition time was 3.67 s, and the delay was 60 s for each sample.

For visualisation, the acquired ^1^H NMR spectra were imported into the NMR software program MestReNova (v14.2.0), while the obtained qNMR spectra for quantification were imported into Bruker's TopSpin NMR software (Version 4.3.0). For all qNMR data, manual phase and baseline correction were conducted and the line broadening applied to all data was 0.1 Hz. For each polymer type, a consistent ppm range was manually integrated across all samples. The integration of the signal areas corresponds to the proton atoms and, consequently, to the concentration of the analyte in the solution. When performing quantitative analysis, care must be exercised in the integration of the signal regions of interest. To minimize potential measurement or integration errors, the internal standard method was employed for all quantitative analyses, as previously published.^28^ The proton signal of DMSO_2_ served as the internal standard across all polymer samples. When utilizing DMSO_2_ as the internal standard, it is essential to ensure that the same concentration is added to each polymer sample and that the concentration is selected to be within a similar intensity range as the deuterated solvents. The concentration of the polymers in their respective solvents was measured as follows (eqn (1)):1
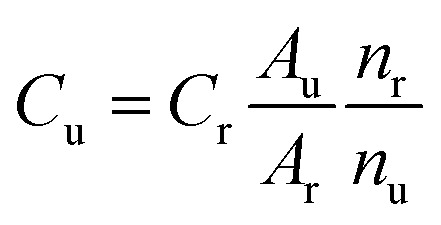
where *C*_u_ is the unknown polymer concentration, *C*_r_ is the known concentration of the internal standard, *A*_r_ is the integral of the proton of the internal standard with known concentration, *A*_u_ is the integral of the proton of the polymer sample with unknown concentration, *n*_r_ is the number of atoms of the proton of the internal standard with known concentration and *n*_u_ is the number of atoms of the proton of the polymer sample with unknown concentration.

### Calculations and statistical analysis

Calculations and statistical analyses were performed, and graphics were made using RStudio (Version 4.3.0). In all setups, each proton signal of each polymer in the different polymer mixtures was measured according to eqn (1) as one replicate sample. For the calibration curve (setup 3A and 3B), polymers with more than one proton signal the total polymer concentration was calculated as the average of the measured concentrations per proton signal. Additionally, for all polymers the concentrations of 125 μg mL^−1^ and 250 μg mL^−1^ were included twice in the calibration curve setup (Table 1, setup 3A and 3B), and were thus used as two data points each. For determining a linear regression, the measured concentrations of the different polymers in the calibration curve setup were plotted against their respective nominal concentrations in micrograms per millilitre (eqn (1)), and a linear regression analysis was run, finding the coefficient of determination (*R*^2^) for each regression line. If *R*^2^ is larger than 0.99, the regression can be considered linear.^35^ The significance level was set to *p* < 0.05 and F-tests were analysed. The upper and lower confidence intervals of 95% were included in the plot.

For setup 1A and 1B, setup 2A and 2B and setup 4 the relative error reports the percent deviation from the nominal concentration. A relative error of 0% means an exact match between the nominal and the measured concentration. The relative error was measured using the formula as follows (eqn (2)):2
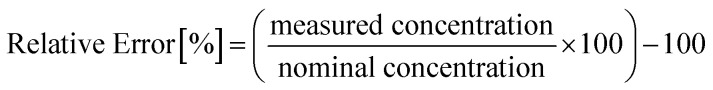


### Prevention of contamination and quality control

To reduce the risk of microplastic contamination from airborne particles, all glassware and other plastic-free equipment were cleaned with water, acetone, and distilled water. All glass flasks were dried for 24 h at 60 °C prior to use and then sealed with a lid once cooled. The NMR tubes were dried for 30 minutes at 60 °C in a closed heating cabinet that had been wiped clean with 95% ethanol before use. Furthermore, laboratory coats composed of pure cotton were used, and samples were covered and kept closed during sampling handling to prevent contamination of polymer fibers. Additionally, nitrile gloves were worn and routinely replaced to avoid cross-contamination. Procedural blanks, consisting of ^1^H NMR and qNMR spectra of pure CDCl_3_ and THF-d_8_ without polymer added, were acquired.

## Results

### Identification – pure polymer ^1^H NMR spectra

To identify the different polymers in the mixtures, pure polymer spectra in their corresponding solvents were prepared and assigned. For clarity, only the regions containing relevant polymer proton signals are considered. The proton signal ranges used for polymer concentration calculations in mixtures are as follows: PS exhibited proton signals in the range of 7.20 to 6.20 ppm (PS-H_a_,H_b_), PB observed proton signals at 5.38 ppm (PB-H_a_) and 2.09 ppm (PB-H_b_), and PI showed proton signals at 5.12 ppm (PI-H_a_) and 2.04 ppm (PI-H_b_). PVC presented a proton signal in the range of 4.70 to 4.25 ppm (PVC-H_a_). For PU, proton signals were identified in the ranges of 8.67 to 8.47 ppm (PU-H_a_,H_e_), 7.43 to 7.29 ppm (PU-H_b_) and 7.07 to 6.98 ppm (PU-H_c_). The spectra and signal assignments are consistent with previously published work^27^ and are provided in Fig. S1 and S2 in the ESI† for reference.

### Quantitative analysis by qNMR spectroscopy of setup 1A and 1B

**Fig. 1:**
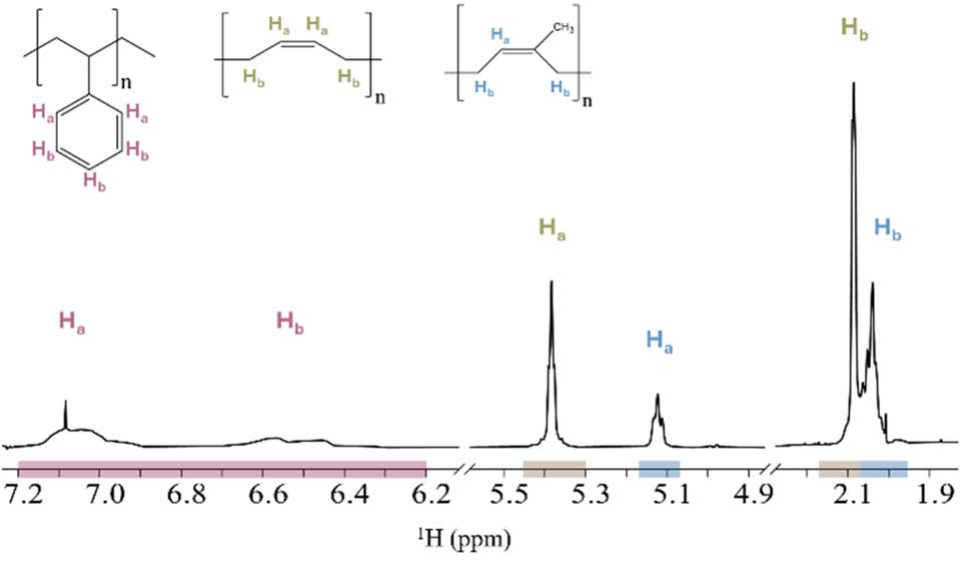
^1^H NMR spectrum and their structural formula of setup 1A (PS, PB and PI in CDCl_3_), showing overlap between signals of PB-H_b_ and PI-H_b_. Integrated areas are highlighted with coloured sections: PS (red), PB (brown) and PI (blue). Proton assignments for each polymer are shown above the corresponding signal areas.

**Fig. 2:**
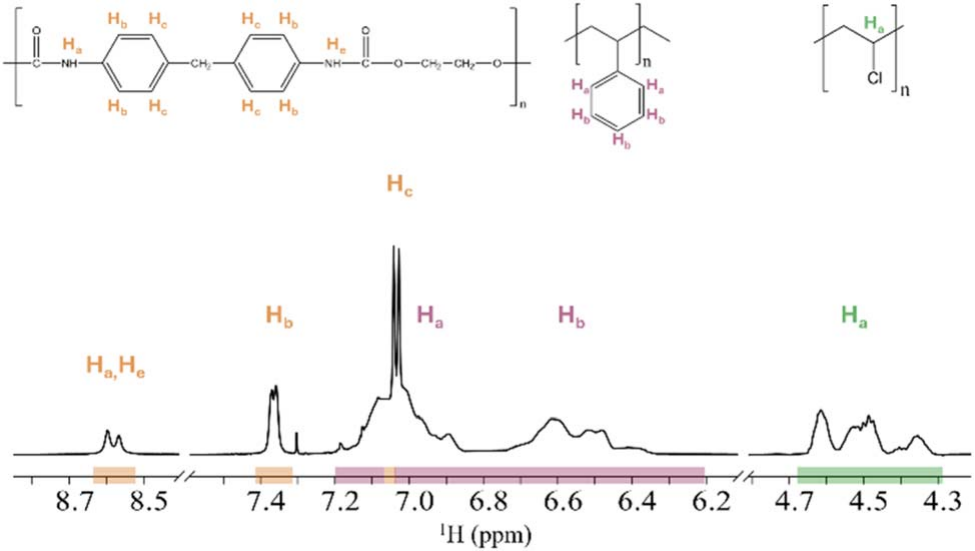
^1^H NMR spectrum and their structural formula of setup 1B (PU, PS and PVC in THF-d_8_), showing overlap between signals of PU-H_c_ and PS-H_a_,H_b_. Integrated areas are highlighted with coloured sections: PU (orange), PS (red), and PVC (green). Proton assignments for each polymer are shown above the corresponding signal areas.

For an overview of the experimental setups, see Table 1. Setup 1A and 1B were prepared with nominal concentrations of 333 μg mL^−1^ for each polymer. The ^1^H NMR spectrum of PS, PB and PI in CDCl_3_ (setup 1A) shows an overlap between the proton signals corresponding to PB-H_b_ and PI-H_b_ (Fig. 1). Similarly, the ^1^H NMR spectrum of PS, PU and PVC in THF-d_8_ (setup 1B) shows an overlap between the proton signals corresponding to PS-H_a_,H_b_ and PU-H_c_ (Fig. 2). Table 2 presents setup 1A and 1B, including the nominal concentrations, the measured concentrations, and their relative error for the respective proton signals of the different polymer types.

**Table 2:** Quantification of the polymer concentrations in mixtures of PS, PB and PI in CDCl_3_ (setup 1A) and PS, PVC and PU in THF-d_8_ (setup 1B). The nominal and measured concentrations [μg mL^−1^], determined using the internal standard method with DMSO_2_, along with the relative error [%] are given for each polymer and setup

Solvent	Polymer type and proton signal	Nominal concentration [μg mL^−1^]	Measured concentration [μg mL^−1^]	Relative error [%]
Setup 1A CDCl_3_	PS-H_a_,H_b_	333	412	24
	PB-H_a_	333	306	−8
	PB-H_b_	333	340	2
	PI-H_a_	333	333	0
	PI-H_b_	333	325	−2
Setup 1B THF-d_8_	PS-H_a_,H_b_	333	381	14
	PVC-H_a_	333	333	0
	PU-H_a_,H_e_	333	134	−60
	PU-H_b_	333	134	−60
	PU-H_c_	333	294	−12

To enable a deeper comprehension of the overlapping signals, the spectrum of the polymer mixture was superimposed with the pure spectra of overlapping polymers. This approach facilitated the identification of the start and end point of the overlapping signals, as well as the degree of their interaction. The overlapping proton signal of PB-H_b_ and PI-H_b_ (setup 1A, Fig. 1) was separated at 2.07 ppm, integrated individually and the concentration measured. The relative errors of PB-H_b_ and PI-H_b_ were +2% and −2%, respectively, using this method. In the overlapping proton signals of PS-H_a_,H_b_ and PU-H_c_ (setup 1B, Fig. 2), PU-H_c_ appears as a sharper signal than PS. Its signal range of 7.07 to 6.98 ppm was used to measure the concentration. The concentration of PS-H_a_,H_b_ was measured based on its proton signals range of 7.20 to 6.20 ppm. The measured concentration of PU-H_c_ was close to its nominal concentration with a relative error of −12%. The measured concentration of PS-H_a_,H_b_ deviated more from its nominal concentration, with relative errors of +24% and +14% in CDCl_3_ and THF-d_8_, respectively. Furthermore, the measured concentrations of the proton signals of PU-H_a_,H_e_ and PU-H_b_ deviated −60% of their nominal concentrations.

Overall, with observed relative error ranges from +24% to −60% for the setups 1A and 1B, the results are not sufficiently consistent for all polymers. This inconsistency depends on solvents and the proton signals used, highlighting the importance of understanding potential interactions and proton signal interferences between polymers in mixtures. It indicates that numbers and integrations directly from the NMR should not be considered accurate and reliable for quantifications when working with mixtures of moderate to high polymer concentrations. Additionally, selecting the appropriate proton signal for polymer measurements is crucial in mixtures.

### Quantitative analysis by qNMR spectroscopy of mixed polymers of different concentrations

#### Polymer mixtures in CDCl_3_ (setup 2A)

**Fig. 3:**
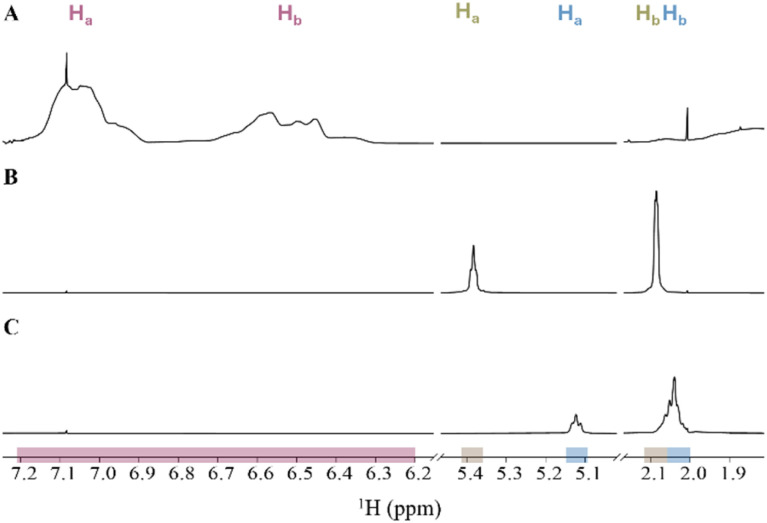
^1^H NMR spectrum of setup 2A with signal ranges for PS (red), PB (brown) and PI (blue) in CDCl_3_: (A) mixture 1: high PS concentration and low PB and PI concentration, (B) mixture 2: high PB concentration and low PS and PI concentration and (C) mixture 3: high PI concentrations and low PS and PB concentrations. Proton assignments for each polymer are shown above the corresponding signal areas.

The ^1^H NMR spectrum of PS, PB and PI in CDCl_3_ (setup 2A) are presented in Fig. 3 and the corresponding quantification data of each proton signal in the different polymer mixtures in Table 3. Mixture 1 using low concentrations of PB and PI show that for the H_a_ proton signal the relative errors of the measured polymer concentrations are very low with +5% and −3%, respectively. Furthermore, the analysis of PS-H_a_,H_b_ gives a relative error of the measured polymer concentration of +20%.

**Table 3:** Quantification of the polymer concentrations in mixtures of PS, PB and PI in CDCl_3_ (setup 2A). The nominal and measured concentrations [μg mL^−1^], determined using the internal standard method with DMSO_2_, along with the relative error [%] are given for each polymer and setup

Solvent CDCl_3_ setup 2A	Polymer type and proton signal	Nominal concentration [μg mL^−1^]	Measured concentration [μg mL^−1^]	Relative error [%]
Mixture 1 (Fig. 3A)	PS-H_a_,H_b_	1000	1196	20
	PB-H_a_	0.4	0.42	5
	PB-H_b_	0.4	8.8	2100
	PI-H_a_	0.6	0.58	−3
	PI-H_b_	0.6	18.4	2967
Mixture 2 (Fig. 3B)	PS-H_a_,H_b_	4	5.7	43
	PB-H_a_	1000	1083	8
	PB-H_b_	1000	1080	8
	PI-H_a_	0.6	4.5	650
	PI-H_b_	0.6	95.6	15 833
Mixture 3 (Fig. 3C)	PS-H_a_,H_b_	4	3.8	−5
	PB-H_a_	0.4	5.3	1225
	PB-H_b_	0.4	187	46 650
	PI-H_a_	1000	1141	14
	PI-H_b_	1000	1007	1

In contrast, in the same mixture we observe an overlap of the proton signals PB-H_b_ and PI-H_b_ (Fig. 3A) which give large deviations from nominal values of +2100% and +2967%, respectively (Table 3). Similarly, we observe that also in mixtures 2 and 3 with high concentration of either PB or PI (Fig. 3B,C) the relative error of measured concentrations for the low-concentration polymer is unacceptable. The short distance between PB-H_a_ and PI-H_a_ affects the proton signal with the lowest concentration the most (Fig. 3B and C). However, the low concentrations of PS in mixtures 2 and 3 gave relative errors of +43% and −5%, respectively.

#### Polymer mixtures in THF-d_8_ (setup 2B)

**Fig. 4:**
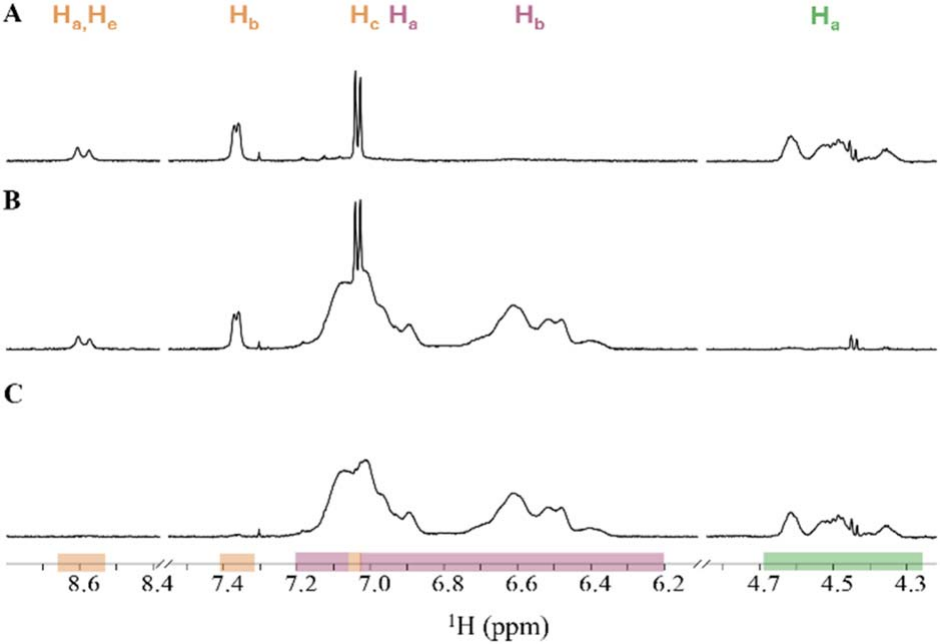
^1^H NMR spectrum of setup 2B with signal ranges for PU (orange), PS (red) and PVC (green) in THF-d_8_: (A) mixture 4: high PU and PVC concentrations and low PS concentration, (B) mixture 5: high PU and PS concentrations and low PVC concentration and (C) mixture 6: high PS and PVC concentrations and low PU concentration. Proton assignments for each polymer are shown above the corresponding signal areas.

The ^1^H NMR spectrum of PS, PVC and PU in THF-d_8_ (setup 2B) are presented in Fig. 4 and the corresponding quantification data of each proton signal in mixtures 4–6 in Table 4. In mixtures 4 and 6 (PVC high) the proton signal of PVC-H_a_ has relative errors of −2% and 4%, respectively (Table 4). In mixture 5 (PVC low) the proton signal of PVC-H_a_ has a relative error of 0%. The results imply that PVC can be safely quantified in both high and low concentrations in mixtures.

**Table 4:** Quantification of the polymer concentrations in mixtures of PS, PVC and PU in THF-d_8_ (setup 2B). The nominal and measured concentrations [μg mL^−1^], determined using the internal standard method with DMSO_2_, along with the relative error [%] are given for each polymer and setup

Solvent THF-d_8_ setup 2B	Polymer type and proton signal	Nominal concentration [μg mL^−1^]	Measured concentration [μg mL^−1^]	Relative error [%]
Mixture 4 (Fig. 4A)	PS-H_a_,H_b_	4	21.0	425
	PVC-H_a_	100	98.0	−2
	PU-H_a_,H_e_	100	35.7	−64
	PU-H_b_	100	35.7	−64
	PU-H_c_	100	40.9	−59
Mixture 5 (Fig. 4B)	PS-H_a_,H_b_	200	221	11
	PVC-H_a_	8	8.03	0
	PU-H_a_,H_e_	100	37.0	−63
	PU-H_b_	100	37.2	−63
	PU-H_c_	100	134	34
Mixture 6 (Fig. 4C)	PS-H_a_,H_b_	200	217	9
	PVC-H_a_	100	104	4
	PU-H_a_,H_e_	6	2.5	−58
	PU-H_b_	6	2.6	−57
	PU-H_c_	6	101	1583

In contrast, we observe that a high concentration of PU and low concentration of PS (Mixture 4) interferes with the proton signals of PS-H_a_,H_b_ (Fig. 4A), and gives a high relative error for both. In the same way as for setup 2A (Fig. 3), the proton signals of PS-H_a_,H_b_ and PU-H_c_ overlap in mixtures 4 and 5 (Fig. 4A and B). In mixtures 5 and 6, the high concentrations of PS-H_a_,H_b_ were measured with acceptable relative errors of 11% and 9%. The slight deviation from nominal values can be due to the overlap with the proton signals of PU-H_c_. However, it is striking that all measured concentrations of PU are below the nominal concentrations. PU-H_a_,H_e_ and PU-H_b_, have unacceptably high relative errors of −64% to −57%. The relative error for the proton signal of PU-H_c_ varies between −59% (Mixture 4) and +1583% (Mixture 6).

### Calibration curves in CDCl_3_ (setup 3A) and THF-d_8_ (setup 3B)

**Fig. 5:**
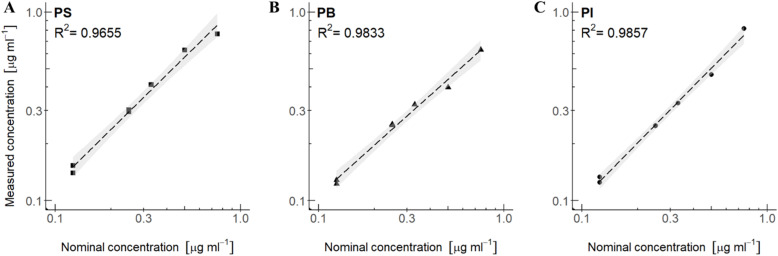
Linear regression of polymer mixtures in CDCl_3_: (A) PS in mixture with PI and PB, (B) PB in mixture with PS and PI and (C) PI in mixture with PS and PB. All polymer mixtures include an internal standard of DMSO_2_ and are represented with a confidence interval (95%). The nominal concentration is plotted against the measured concentration (measured by eqn (1)).

**Fig. 6:**
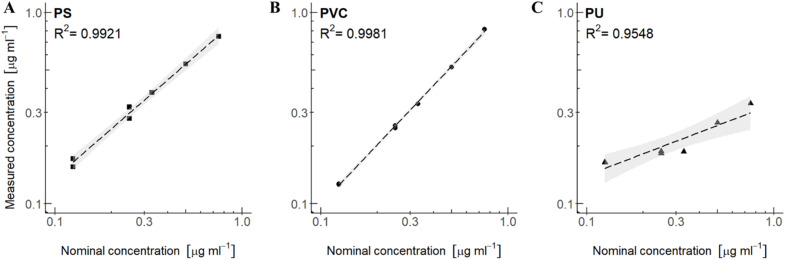
Linear regression of polymer mixtures in THF-d_8_: (A) PS in mixture with PVC and PU (B) PVC in mixture with PS and PU and (C) PU in mixture with PS and PVC. All polymer mixtures include an internal standard of DMSO_2_ and are represented with a confidence interval (95%). The nominal concentration is plotted against the measured concentration (measured by eqn (1)).

In the calibration curve (setup 3A and 3B) concentrations of the different polymers in mixtures were analysed and plotted against the nominal concentrations. To find the measured concentration of polymers with multiple proton signals, the concentrations measured from each proton signal were averaged. When there were two data points for the same concentration (125 μg mL^−1^ and 250 μg mL^−1^ of each polymer) in mixtures, the two data points were used as two replicates for the same concentration (Fig. 5 and 6).

For setup 3A, polymer concentration mixtures in CDCl_3_ of PS in mixture with PB and PI give an *R*^2^ of 0.9655 (Fig. 5A), PB in mixture with PS and PI give an *R*^2^ of 0.9833 (Fig. 5B), and PI in mixture with PS and PB give an *R*^2^ of 0.9857 (Fig. 5C). Although the *R*^2^ values are below 0.99, it still implies a good linear relationship between measured concentrations and nominal concentrations in the range of concentrations that were tested. For setup 3B, polymer mixtures in THF-d_8_, the measured *versus* nominal concentrations give good *R*^2^ values for the polymers PS and PVC. *R*^2^ for PS in mixture with PVC and PU is 0.9921 (Fig. 6A) and PVC in mixture with PS and PU has an *R*^2^ of 0.9981 (Fig. 6B), implying a good linear relationship between measured concentrations and nominal concentrations for these polymer mixtures. PU in mixture with PS and PVC, however, gives an *R*^2^ of 0.9548 (Fig. 6C), which corresponds with the large relative error observed for PU in mixtures (Table 4). In both setups, linear regression analysis (*cf.* the ESI†) revealed significance levels of *p* < 0.05 and high F-test values of each polymer type, indicating a significant relationship between the measured and nominal concentrations.

In contrast to setup 2A and 2B with concentration spans from 0.4 to 1000 μg mL^−1^, the concentration span among polymers in the calibration curve are less pronounced, and we also observe a higher precision in the measurements. These spans may describe realistic concentration ratios in environmental samples, and the results indicate that the method is able to quantify the polymers in these mixtures with good accuracy except for PU.

### Quantitative analysis by qNMR spectroscopy of mixed polymers in THF-d_8_

**Fig. 7:**
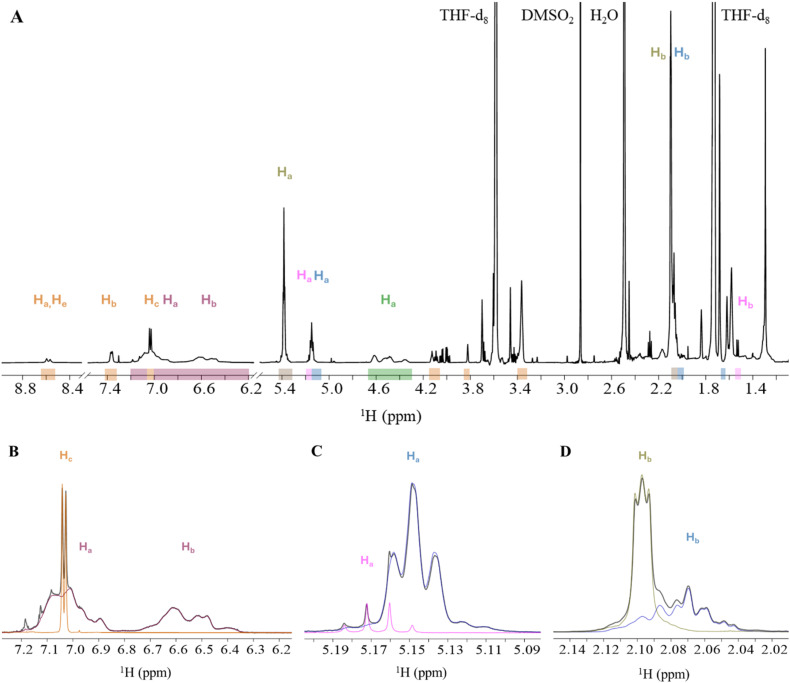
(A) Full ^1^H NMR spectrum of setup 4 with signal ranges for PU (orange), PS (red), PB (brown), PLA (pink), PI (blue), and PVC (green) in THF-d_8_. Panels B–D: detail showing three areas with overlapping polymer signals (black spectral line) of (B) PS-H_a_,H_b_ and PU-H_c_, (C) PLA-H_a_ and PI-H_a_ and (D) PB-H_b_ and PI-H_b_ with the same colour coding as in (A). Proton assignments for each polymer are shown above the corresponding signal areas.

To facilitate a more comprehensive examination of the potential for detecting and quantifying multiple polymers in mixtures, in the third part a sample was prepared by combining equal volumes of stock solutions containing the six polymers PS, PVC, PI, PB, PU, and PLA in THF-d_8_ (Fig. 7A). Each polymer had a nominal concentration of 166 μg mL^−1^ as shown in Table 1. The ^1^H NMR spectrum of the six-polymer-mixture in THF-d_8_ shows additional signals beyond those previously utilized. These signals have not previously been used for concentration calculations, and were not used in this work either, as the low ppm ranges are in regions where signals from water and DMSO_2_ and other impurities of the solvents are also observed.^36^ These omitted signal ranges include further proton signals of PU in the range of 4.15 to 4.95 ppm and a signal at 3.36 ppm, as well as a proton signal at 3.82 ppm. Additionally, a third signal of PI (PI-H_c_) was observed at 1.68 ppm and two proton signals of PLA were detected in the ranges of 5.20 to 5.12 ppm (PLA-H_a_) and 1.61 to 1.56 ppm (PLA-H_b_), as previously assigned.^27^ The concentrations of these three proton signals in the polymer mixture (setup 4) were measured according to eqn (1) and are shown in Table 5.

**Table 5:** Quantification of the polymer concentration in the six-polymer-mixture of PS, PB, PI, PVC, PU and PLA in THF-d_8_ (setup 4). The nominal and measured concentrations [μg mL^−1^], determined using the internal standard method with DMSO_2_, along with the relative error [%] are given for each polymer and setup

Polymer type and proton signal	Nominal concentration [μg mL^−1^]	Measured concentration [μg mL^−1^]	Relative error [%]
PS-H_a_,H_b_	166	204.6	23
PB-H_a_	166	167.2	1
PB-H_b_	166	198.7	20
PI-H_a_	166	159.0	−4
PI-H_b_	166	107.7	−35
PI-H_c_	166	155.2	−7
PVC-H_a_	166	174.4	5
PU-H_a_,H_e_	166	70.2	−58
PU-H_b_	166	70.6	−57
PU-H_c_	166	154.9	−7
PLA-H_a_	166	167.1	1
PLA-H_b_	166	26.6	−84

As previously observed in setups 1 and 2, the proton signals of PS-H_a_,H_b_ and PU-H_c_ (Fig. 7B), as well as those of PB-H_b_ and PI-H_b_ (Fig. 7C), were observed to overlap in this six-polymer-mixture as well. Additionally, the proton signals of PLA-H_a_ and PI-H_a_ (Fig. 7D) were observed to overlap.

The quantitative analyses of individual proton signals for the various polymers indicate that the measured concentrations of PB-H_a_, PI-H_a_, PI-H_c_, PVC-H_a_, PU-H_b_, PU-H_c_ and PLA-H_a_ align closely with the nominal concentration of the polymers (Table 5), with relative errors form −7% to +1%. In contrast, measured concentrations from the overlapping proton signals of PS-H_a_,H_b_ with PU-H_c_, as well as the proton signals of PB-H_b_, PI-H_b_, PU-H_a_,H_e_ and PU-H_b_, gave polymer concentrations significantly different from their nominal concentrations (Table 5), with relative errors in the range from −58% to +23%. Additionally, although not overlapping with any other proton signal, the measured concentration of PLA-H_b_, in low ppm range, differs substantially from the nominal concentration, and has a relative error of −84%.

## Discussion

Recent studies on the use of NMR spectroscopy in the field of micro- and nanoplastics have advanced the understanding of how the method may be applied but has also shown that the use of qNMR spectroscopy is a method that still requires further development for the quantification of certain polymers. The method is rapid, reliable and cost-efficient, but challenges arise with mixtures, which is particularly relevant for environmental samples. Previous research has focused on examining pure polymers individually, and only a few have investigated polymer mixtures.^33,34^

### Challenges with high and low concentrations in a mixture

The current study used polymer mixtures of three different polymers in two solvents in varying concentration setups, ranging from 8 to 0.4 μg mL^−1^, based on previously published concentration limits for single polymers.^27^ The current investigation showed that the different concentration ratios influenced the determination of the respective polymer proton signals. The polymer mixtures containing equal parts of high concentrations showed that proton signals of PB and PI in CDCl_3_ and PS and PU in THF-d_8_ overlapped, a problem that persisted in the quantitative determination of the polymers in mixtures containing high and low concentrations. The overlap of the proton signals as well as the concentration ratios in the mixture can complicate an exact quantitative analysis of the overlapping polymers. To our knowledge, this study is the first to investigate overlapping proton signals and their effect on quantification, using the internal standard method. The simultaneous quantification of two polymer mixtures: one containing PVC, PS and BR, and the other containing PET and PA, using various non-deuterated solvent systems along with an internal standard, was previously reported.^34^ The study used two concentration ranges of 2.5 to 0.5 mg mL^−1^ and 0.5 to 0.1 mg mL^−1^, significantly higher than those in this work. The calibration curve was based on LOD and LOQ values determined using DIN 32645 and SNR and the polymers in the mixtures were measured based on this calibration method.^34^ Results showed accurate quantification of these polymers down to 0.1 mg mL^−1^, achieving high linearity (*R*^2^ above 0.99) and precision in the calibration curves. The high concentration ranges utilized may not be representative of lower concentrations typically encountered in real environmental samples.^34^

### Challenges with quantifying multiple polymers in a mixture

The polymer mixture of PS, PVC, PU, PB, PI and PLA in THF-d_8_ provided a good overview of the identification and quantification of the various polymer proton signals. To our knowledge, this is the first study to use qNMR quantification to identify and accurately quantify six different polymers in one solvent and in one sample at concentrations down to 166 μg mL^−1^ using qNMR spectroscopy.

A selective separation of eight polymers from inorganic components, along with the simultaneous fractionation of different polymer types before the use of qNMR analysis, was presented in recent research.^33^ This approach tested a set of eight relevant polymers (PS, BR, PVC, PET, PA, LDPE, PMMA and PAN) dissolved sequentially in four different non-deuterated solvents (THF, trifluoroacetic acid in chloroform, formic acid in chloroform and xylene) in a concentration range of 2.5 to 0.5 mg mL^−1^, and showed that separating different polymer types is essential for qNMR analysis of polymers, as variable solubilities require a reliable fractionation procedure to thoroughly analyse the diverse range of polymers present in environmental matrices. The study enabled the quantification of the polymers PS, PVC, PET, PA and PMMA from a single sample and observed recovery rates of over 88% for all tested polymers, whereas LDPE and PAN were absent in the NMR spectra.^33^ Moreover, the lower concentrations observed and expected in real environmental samples may not be accurately reflected by the high concentrations range used. The duration of the extraction process can vary depending on whether the complete set of polymers requires analysis, with the full procedure potentially being completed within a timeframe of approximately 3 to 4 hours. The use of non-deuterated solvents for the extraction process can help maintain the costs of this procedure at a lower level compared to the utilization of deuterated solvents.^33^

### Overlapping proton signals in qNMR spectroscopy of mixtures

The overlapping proton signals of the polymers in different concentration ratios represent a challenge in the accurate quantification of polymers in mixtures, which is highly relevant for environmental samples, where varying concentrations must also be expected. When a polymer has a distinct, single signal, it is preferable to be use that signal for quantification. In this study, overlapping proton signals were identified using the pure proton spectra of individual polymers. If no single signals are available, deconvolution in qNMR spectroscopy for polymer mixtures could help to resolve overlapping signals, enabling quantification of individual components despite their chemical similarities, even in complex samples. However, this approach faces certain challenges, such as the presence of broad peaks, noise, baseline distortions, and the requirement for accurate peak shape assumptions and reference data, which may potentially limit the reliability and broader applicability of the technique.^37,38^ Based on experience from this study, deconvolution did not produce spectra that resembled the pure spectra and the quantified concentrations deviated from the nominal values (Table S2 and Fig. S3 and S4 in the ESI†). Consequently, we do not recommend using deconvolution in polymer mixtures with broad, overlapping signals or highly varying concentrations. Additionally, in cases of overlapping proton signals from different polymers, lower concentrations of one polymer can remain undetected due to signal interference from other polymers with higher concentrations, complicating accurate quantification in complex polymer mixtures. Overall, while the internal standard method is generally more reliable for accurate quantification, particularly for low concentrations and signal overlap, deconvolution remains less accurate due to its limitations with broad signals. Nevertheless, it may be useful in certain cases when prior knowledge of signal appearance is available.

### Solvents and internal standards

The use of the two solvents showed that the use of THF-d_8_ was preferable to CDCl_3_. THF-d_8_ allowed the dissolution of six different polymers that could be analysed simultaneously in a single sample, additionally avoiding the collapsing between the right ^13^C satellite of CDCl_3_ at 7.08 ppm with the proton signal of PS (*e.g.*Fig. 1A).^39^ Nevertheless, identifying suitable solvents for a wide range of polymers, or utilizing different solvents to target various polymer types and avoid signal overlap, may be essential for the successful application of qNMR spectroscopy in the analysis of polymer mixtures. Furthermore, the use of internal standards needs to be carefully chosen when analysing polymer mixtures containing several different polymers or even environmental samples. In this study, DMSO_2_, with a proton signal at 3.00 ppm, was used as the internal standard. From previous studies, it is known in which ppm ranges the different proton signals of the chosen polymers appear,^22–24,27,33^ but the use of DMSO_2_ in an unknown environmental sample could lead to overlapping signals and loss of information about unexpected polymers or polymers with low concentrations. Hexamethyldisiloxane (HMDSO), with a proton signal at 0.25 ppm, proved to be suitable as an internal standard.^33,34^

### Future research needs

Considering the additional use of deconvolution of proton signals, qNMR spectroscopy may serve as a cost-effective, rapid and reliable method for identifying and quantifying various polymers in a mixture, enabling the quantification of several different polymers in a single sample.

As the environmental concern over environmental plastic and microplastic pollution increases, and in the likely event of a global plastic treaty, management and control with release of plastic pollution through wastewater, as well as overall monitoring of environmental levels may become mandatory. A rapid and reliable method for quantification of common polymers in a range of matrices may thus be in high demand. Currently, qNMR spectroscopy could be used for the quantification of plastic polymers in highly concentrated water, sediment or soil samples in environmental compartments such as highly polluted rivers or sea-floor sediments from urban areas, or in municipal or industrial wastewater.^40,41^ Hence, besides the relevant research on pristine polymer mixtures,^33,34^ studies using realistic environmental samples with polymers that have been aged and weathered are also required. Since UV-light and heat cause degradations in polymers through chain scission, oxidation, crosslinking, and loss of side groups, these changes may manifest in ^1^H NMR as new peaks, altered chemical shifts, peak broadening, and decreased intensity of original signals.^42,43^ Hence, NMR spectroscopy can serve as a tool to monitor and analyse the structural changes in polymers resulting from aging and weathering (Schmidt *et al.*, in prep). Moreover, consideration must be given to ensure that environmental samples go through a thorough sample preparation (extraction, filtration and purification) prior to NMR spectroscopy to avoid further potential overlaps with inorganic and organic compounds, whose proton signals appear in the lower ppm ranges (<3 ppm).^24^

Another consideration is the relevance of the polymers that we can quantify so far. Since the dissolution of polymers currently limits the use of NMR spectroscopy, further research is necessary to find methods and solvents that make common polyolefins such as polypropylene (PP) and polyethylene (PE) more suitable for NMR spectroscopy. These polymers, along with PS, PVC and PU, represent the largest proportion of plastic production, and thus, the most prevalent in the environment,^1,44^ hence the demand for reliable monitoring tools. The quantification of polyolefins in polymer mixtures using qNMR spectroscopy remains a challenge that has not yet been fully overcome.^33^ However, rubber-based polymers such as PB and PI, which originate from tire wear production, and the bioplastic PLA, which derives from renewable biomass sources, are becoming increasingly environmentally relevant polymers.^45–47^

## Conclusions

This study highlights the potential of qNMR spectroscopy for quantifying mixed polymer samples, offering critical insights into its application for complex environmental samples. Polymer mixtures of PS, PB, and PI in CDCl_3_ show that low concentrations of PS can be effectively quantified by qNMR spectroscopy, with a relative error of −5%, when PS is present in both high and low concentrations. In the polymer mixtures, PB and PI can be quantified down to their previously measured concentration limits for single polymers, using their first proton signal (H_a_) at low concentrations, with relative errors of +5% and −3%, respectively. If one of these polymers is present at a high concentration, it can interfere with the detection and quantification of the other polymer at a lower concentration, compromising measurement accuracy. Additionally, the overlapping signal ranges for the second proton signals (H_b_) of PB and PI make these signals unsuitable for quantifying their concentrations.

Analysis of polymer mixtures containing PS, PVC and PU, dissolved in THF-d_8_, demonstrate that PVC concentrations can be reliably quantified in low concentration mixtures with a relative error of 0% and high concentration mixtures with relative errors of −2% and 4%. In contrast, the quantification of the measured concentration limit of PS varies from the nominal concentration due to the overlap with a proton signal of PU. Therefore, the measured concentrations of PU diverged severely from the nominal concentrations, and the third proton signal (H_c_) especially seems to be less reliable basis for concentration calculations in mixtures.

Linear regression analyses in CDCl_3_ demonstrated strong correlation (*R*^2^ below 0.99) between the measured and nominal concentrations within the tested concentration range, while in THF-d_8_, de, PS and PVC achieved excellent correlations (*R*^2^ above 0.99), contrasting with PU's reduced correlation (*R*^2^ = 0.9548). These findings underscore the importance of signal separation and solvent choice in achieving accurate quantification, particularly in complex polymer mixtures. Further refinement of qNMR methods is necessary to overcome the limitations posed by signal overlaps and to enhance its applicability to environmental sample analysis.

## Data availability

The data supporting this article have been included as part of the ESI.†

## Author contributions

J. S. designed the methodology, conducted the investigation, curated and analyzed the data, and wrote the original draft. J. S., M. H., and W. W. reviewed and edited the manuscript. J. S. and M. H. contributed to visualization. W. W administered the project and acquired funding.

## Conflicts of interest

There are no conflicts to declare.

## Supplementary Material

RA-015-D5RA01174D-s001
