# Supplementary material for: Applicability of NMR spectroscopy to quantify microplastics across varying concentrations in polymer mixtures
Source: RSC Adv. 2025 Apr 23;15(17):13041–52. doi: 10.1039/d5ra01174d (PMC12016022; doi:10.1039/d5ra01174d)
Supplement: RA-015-D5RA01174D-s001 [file RA-015-D5RA01174D-s001.pdf]

Supplementary Information (SI) for RSC Advances.
This journal is © The Royal Society of Chemistry 2025

Supporting Information

Applicability of NMR spectroscopy to Quantify Microplastics Across Varying Concentrations in Polymer Mixtures

Julia Schmidt[1], Marte Haave[3], Wei Wang[1,2*]

[1] Department of Chemistry, University of Bergen, 5007 Bergen, Norway

[2] Centre for Pharmacy, University of Bergen, 5020 Bergen, Norway

[3] SALT Lofoten AS, 8301 Svolvær, Norway

* Corresponding Author: Wei Wang, wei.wang@uib.no

Identification of polymer proton signals of pure microplastic ^{1}H NMR spectra

The single spectra of PS, PB and PI in $CDCl_3$ (Fig. S1) and PS, PVC and PU in THF-d_8 (Fig. S2) show their proton signals relevant for this study and later used for calculations of polymer concentrations in mixtures. For illustration purposes, only the regions with relevant polymer proton signals are shown. The proton signal ranges of the different polymers relevant to this study are summarized as: PS exhibited signals between 7.20 and 6.20 ppm (Fig. S1A and S2A), PB at 5.38 ppm and 2.09 ppm (Fig. S1B), and PI at 5.12 ppm and 2.04 ppm (Fig. S1C). PVC displayed a signal between 4.70 and 4.25 ppm (Fig. S2B), while PU showed signals in the ranges of 8.67–8.47 ppm, 7.43–7.29 ppm, and 7.07–6.98 ppm (Fig. S2C).

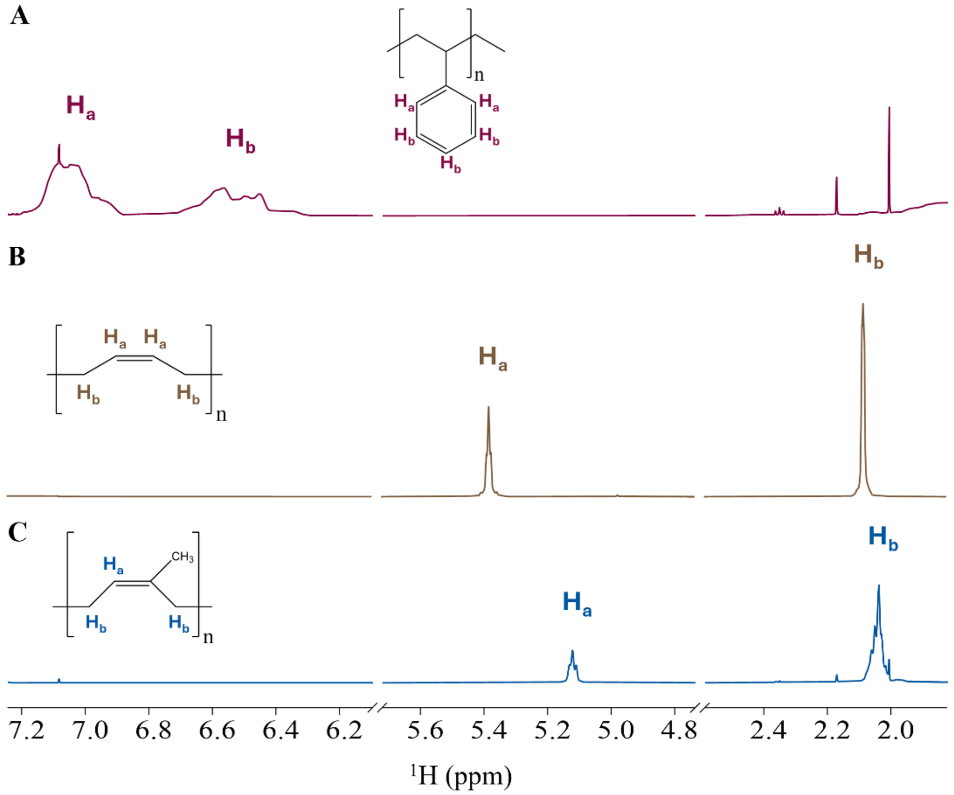

Fig. S1 ^{1}H NMR spectrum and their structural formula of (**A**) polystyrene (PS), (**B**) polybutadiene-cis (PB) and (**C**) polyisoprene-cis (PI) in $CDCl_3$ with a nominal polymer concentration of 1 mg mL^{-1}.

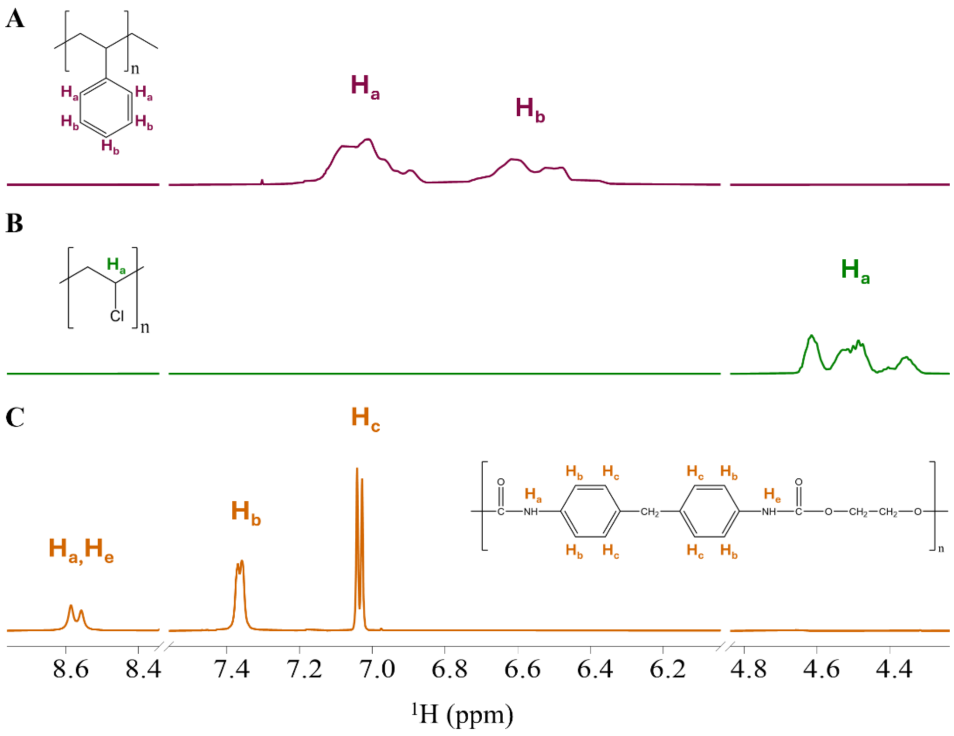

Fig. S2 ^{1}H NMR spectrum and their structural formula of (**A**) polystyrene (PS), (**B**) polyvinyl chloride (PVC) and (**C**) polyurethane (PU) in THF-d_8 with a nominal polymer concentration of 1 mg mL^{-1}.

Linearity data

To ensure the linearity of the calibration curve data, the coefficient of determination (R^2), slope, and intercept of the linear regression for each polymer were examined. Additionally, to assess whether there is a statistically significant relationship between the measured concentration and the nominal concentration in the calibration curves (Setup 3A and 3B), an F-test for regression was performed.

Table S1 Overview of linearity data for the different polymers in their calibration curve. The slope coefficient, intercept, coefficient of determination (R^2), F-test, and p-value are provided.

	**Polymer type and proton signal**	**Slope**	**Intercept**	R^2	**F-test**	***p*-value**
**$CDCl_3$ Setup 3A**	PS - H_a,H_b	0.93958	-0.02888	0.9655	169.1	4.795e-05
	PB - H_a/H_b	1.27145	-0.04950	0.9833	355	7.759e-06
	PI - H_a/H_b	0.92515	0.01996	0.9857	413.3	5.294e-06
**THF-d_8 Setup 3B**	PS - H_a,H_b	1.05934	-0.05970	0.9921	756.4	1.189e-06
	PVC - H_a	0.91004	0.01772	0.9981	3098	3.54e-08
	PU - H_a,H_e/ H_b/ H_c	3.46261	-0.40220	0.9548	127.8	9.47e-05

PS = polystyrene, PB = polybutadiene-cis, PI = polyisoprene-cis, PVC = polyvinyl chloride, PU = polyurethane

Deconvolution

To test quantification of overlapping signals using deconvolution, the overlapping signals of PB-H_b and PI-H_b in $CDCl_3$ and PS-H_a,H_b and PU-H_c in THF-d_8 in the setups 1 and 2 were analysed using the NMR software program MestReNova (v14.2.0). The polymer concentrations were measured using Equation 1 and the relative error using Equation 2. Results are shown in Table S2.

In cases where the polymers are present at equal concentrations, deconvolution performs as well as the internal standard method (Fig. S3A). However, when both PB and PI are at low concentrations, neither method is suitable due to interference from the high concentration of PS, which causes significant signal overlap (Fig. S3B). For mixtures with high PB and low PI concentrations, or high PI and low PB concentrations, both methods successfully quantify the higher concentrations (Fig. S3C,D). However, the measured values for the lower concentrations deviate considerably from the nominal values. While deconvolution provides lower relative errors (e.g., 1484 % for PB-H_b) compared to the internal standard method (e.g., 46650 % for PB-H_b), the results remain highly inaccurate. Additionally, the deconvolution benefited from prior knowledge of the appearance of the PB-H_b and PI-H_b signals at low concentrations, which was essential for distinguishing between them and helped guide its use for more accurate analysis in this case.

Overall, neither method is ideal for accurately measuring low concentrations of the overlapping PB-H_b and PI-H_b signals. Therefore, it is recommended to use the distinct, single signals of PB-H_a and PI-H_a for quantification.

Table S2 Overview of the quantitative results from the deconvolution method compared to those obtained using the internal standard method. The analysis focuses on the overlapping signals of PB-H_b and PI-H_b in $CDCl_3$ and PS-H_a,H_b and PU-H_c in THF-d_8 in polymer mixtures for setups 1 and 2. Nominal concentrations (C_n) and measured concentrations (C_m) are presented in µg mL^{-1}, calculated using Equation 1, along with the relative error (RE) [%] determined using Equation 2.

				**Deconvolution**		**Internal standard method**	
			C_n [µg mL^{-1}]	C_m [µg mL^{-1}]	RE [%]	C_m [µg mL^{-1}]	RE [%]
**$CDCl_3$**	Setup 1A	PB-H_b	333	329	-1	340	2
		PI-H_b	333	309	-7	325	-2
	Setup 1B - Mixture 1	PB-H_b	0.4	NA	NA	8.8	2100
		PI-H_b	0.6	NA	NA	18.4	2967
	Setup 1B - Mixture 2	PB-H_b	1000	1007	1	1083	8
		PI-H_b	0.6	14	2269	95.6	15833
	Setup 1B - Mixture 3	PB-H_b	0.4	6	1484	187	46650
		PI-H_b	1000	846	-15	1007	1
**THF-d_8**	Setup 1B	PS-H_a,H_b	333	268	-20	381	14
		PU-H_c	333	184	-45	294	-12
	Setup 2B - Mixture 4	PS-H_a,H_b	4	9	119	21	425
		PU-H_c	100	44	-56	41	-59
	Setup 2B - Mixture 5	PS-H_a,H_b	200	177	-11	221	11
		PU-H_c	100	61	-39	134	34
	Setup 2B - Mixture 6	PS-H_a,H_b	200	205	3	217	9
		PU-H_c	6	NA	NA	101	1553

PS = polystyrene, PB = polybutadiene-cis, PI = polyisoprene-cis, PVC = polyvinyl chloride, PU = polyurethane

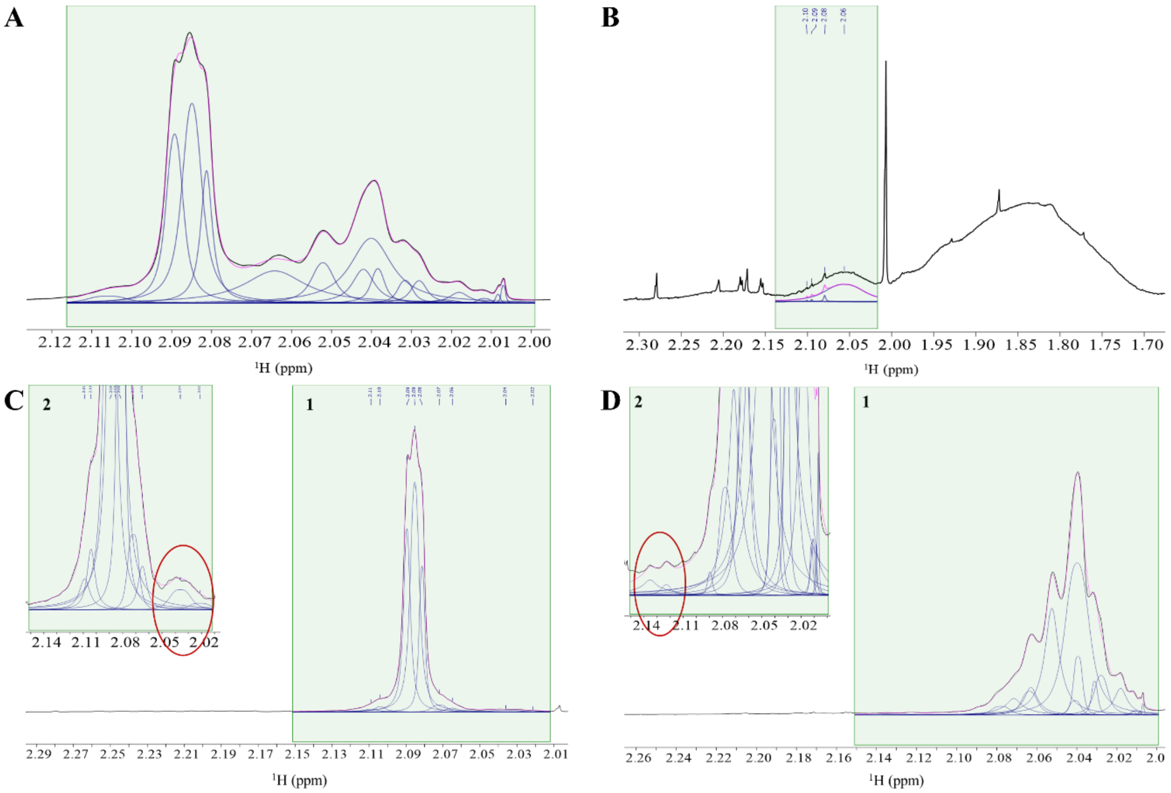

Fig. S3 ^{1}H NMR spectra showing the deconvolution of overlapping signals from PB-H_b and PI-H_b in polymer mixtures in $CDCl_3$: (**A**) Setup 1A with equal concentration levels, (**B**) Setup 2A - Mixture 1 with low PI and PB concentrations and high PS concentration, (**C**) Setup 2A - Mixture 2 with low PS and PI concentrations and high PB concentration (with the red circle highlighting the PI-H_b signal), and (**D**) Setup 2A - Mixture 3 with low PS and PB concentrations and high PI concentration (with the red circle highlighting the PB-H_b signal). Panel C and D include both a larger overview (2) and a detailed view (1). Each panel displays the peak curve (blue) and the sum curve (purple).

When the two polymers of PS and PU are present at equal concentrations (Fig. S4A), the internal standard method provides more accurate results, with better relative errors compared to deconvolution. However, when PS is at a low concentration and the concentration of PU is high (Fig. S4B), deconvolution appears to yield better quantitative results for PS. Yet, a closer examination reveals that parts of the broad PS signal were not accounted for, as the model failed to detect the area in the ppm range of 7.02 to 6.98, leading to an underestimated concentration(Fig. S4 A,B). For PU, both methods produce similar results at high concentrations.

When both PS and PU are present at high concentrations (Fig. S4C), the two methods perform comparably. However, deconvolution tends to underestimate the PS concentration, likely due to the missing signal area between 7.02 and 6.92 ppm (Fig. S4C). In contrast, in mixtures where the concentration of PS is high and the concentration of PU is low (Fig. S4D), both methods provide good

results for PS, with deconvolution achieving a better relative error. Meanwhile, the low PU concentration cannot be reliably measured by either method, as its signal is completely masked by the high PS concentration.

Overall, while both methods perform well in certain scenarios, neither is ideal for accurately quantifying low PU concentrations in the presence of high PS. Deconvolution, in particular, has limitations with broad PS signals, which reduce its accuracy, and therefore, it is not recommended for the quantification of broad signals in polymer mixtures.

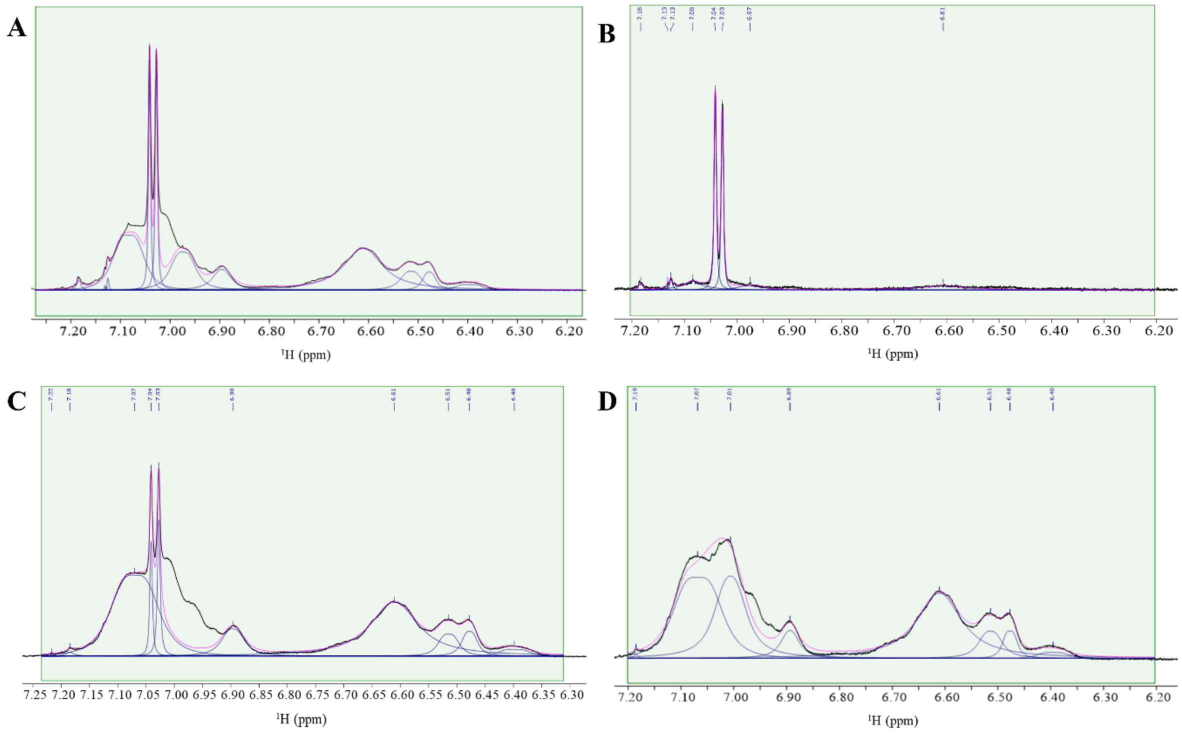

Fig. S4 ^1H NMR spectra showing the deconvolution of overlapping signals from PS-H_a,H_b and PU-H_c in polymer mixtures in THF-d_8: (**A**) Setup 1B with equal concentration levels, (**B**) Setup 2B - Mixture 4 with low PS concentration and high PU and PVC concentrations, (**C**) Setup 2B - Mixture 5 with low PVC concentration and high PS and PU concentrations, and (**D**) Setup 2B - Mixture 6 with low PU concentration and high PS and PVC concentrations. Each panel displays the peak curve (blue) and the sum curve (purple).
